# The mixture of cashew nut shell liquid and castor oil results in an efficient larvicide against *Aedes aegypti* that does not alter embryo-fetal development, reproductive performance or DNA integrity

**DOI:** 10.1371/journal.pone.0193509

**Published:** 2018-03-19

**Authors:** Juliana Miron Vani, Maria Tereza Ferreira Duenhas Monreal, Sarah Alves Auharek, Andréa Luiza Cunha-Laura, Eduardo José de Arruda, Alessandra Ramos Lima, Cicera Maria da Silva, Andréia Conceição Milan Brochado Antoniolli-Silva, Dênis Pires de Lima, Adilson Beatriz, Rodrigo Juliano Oliveira

**Affiliations:** 1 Centro de Estudos em Células Tronco, Terapia Celular e Genética Toxicológica—CeTroGen, Hospital Universitário Maria Aparecida Pedrossian–HUMAP, Universidade Federal de Mato Grosso do Sul—UFMS, Campo Grande, MS, Brasil; 2 Programa de Mestrado em Farmácia, Faculdade de Ciências Farmacêuticas Alimentos e Nutrição—FACFAN, Universidade Federal de Mato Grosso do Sul–UFMS, Campo Grande, MS, Brasil; 3 Programa de Pós-graduação em Saúde e Desenvolvimento na Região Centro-Oeste, Faculdade de Medicina “Dr. Hélio Mandetta”–FAMED, Universidade Federal de Mato Grosso do Sul–UFMS, Campo Grande, MS, Brasil; 4 Faculdade de Medicina do Mucuri, Universidade Federal dos Vales do Jequitinhonha e do Mucuri–UFVJM, Teófilo Otoni, MG, Brasil; 5 Faculdade de Ciências Exatas e Tecnologia–FACET, Universidade Federal da Grande Dourados–UFGD, Dourados, MS, Brasil; 6 Instituto de Química—INQUI, Universidade Federal de Mato Grosso do Sul–UFMS, Campo Grande, MS, Brasil; 7 Programa de Pós-graduação em Genética e Biologia Molecular, Centro de Ciências Biológicas–CCB, Universidade Estadual de Londrina, Londrina, PR, Brasil; CEA, FRANCE

## Abstract

Dengue fever, chikungunya fever and Zika virus are epidemics in Brazil that are transmitted by mosquitoes, such as *Aedes aegypti* or *Aedes albopictus*. The liquid from shells of cashew nuts is attractive for its important biological and therapeutic activities, which include toxicity to mosquitoes of the genus *Aedes*. The present study evaluated the effects of a mixture of surfactants from natural cashew nutshell liquid and castor oil (named TaLCC-20) on the mortality of larvae and on the reproductive performance, embryonic and fetal development and genetic stability of Swiss mice. A total of 400 *Ae*. *aegypti* larvae (third larval stage) were treated with TaLCC-20 concentrations of 0.05 mg/L, 0.5 mg/L, or 5 mg/L (ppm). Twenty pregnant female mice were also orally administered TaLCC-20 at doses of 5 mg/kg and 50 mg/kg body weight (b.w.), and 10 animals were given only drinking water at 0.1 mL/10 g b.w. (orally). The results of a larvicide test demonstrated that 5 mg/mL TaLCC-20 killed 100% of larvae within three hours, which is comparable to the gold standard indicated by the Ministry of Health. Overall, these results show that TaLCC-20 is an efficient larvicide that does not induce genetic damage. In addition, changes in reproductive performance and embryo-fetal development appear positive, and the formulation is cost effective. Therefore, TaLCC-20 is an important product in the exploration of natural larvicides and can assist in fighting mosquitos as vectors for dengue fever, chikungunya fever and Zika virus, which are emerging/re-emerging and require proper management to ensure minimal harm to the human population. Therefore, TaLCC-20 can be considered a key alternative to commercial products, which are effective yet toxigenic.

## Introduction

Dengue is an epidemic in Brazil and worldwide. Coupled with chikungunya fever and Zika virus, these three diseases have caused extensive public health problems. In 2016, there were approximately 1,426,005 probable cases of dengue, including 798 severe cases and 7,105 suspected cases. In addition, dengue was responsible for 509 deaths [1].

In Brazil, 3,657 cases of chikungunya fever were diagnosed in 2014. In 2016, the number of cases increased to 216,102 autochthonous cases. Zika virus was first diagnosed in Brazil in 2015, when 1,248 cases were reported in the northeast, in addition to 739 cases of microcephaly in newborns, which characterizes the most serious form of the disease. In 2016, there were approximately 196, 976 probable cases of the disease [1–3].

Transmission of these diseases occurs through the mosquitoes *Aedes aegypti* and *Aedes albopictus* after a bite from an infected female. There is currently no effective vaccine for these diseases. Thus, the prevention of these diseases depends exclusively on the elimination of mosquito foci [4–6].

According to Machado et al. [7], the most promising results recorded to date regarding the prevention of increasing cases of dengue, chikungunya fever and Zika virus in Brazil were obtained with the use of diflubenzuron and, more recently, with pyriproxifen. Therefore, these are the compounds of choice indicated by the Brazilian Ministry of Health for use in fumigation and deposition in areas containing standing water, including water tanks used for human consumption. However, this practice is not recommended and should be discontinued, as the consumption of diflubenzuron causes genetic/genomic instability and increases predisposition to chronic diseases, such as cancer [8]. For pyriproxifen, there are no available studies on its mutagenicity and teratogenicity in the literature.

Accordingly, there is a critical need for new larvicidal compounds that do not exhibit toxicity to humans or the environment. One attractive organic raw material is cashew nut (*Anarcadium occidentale* L.) shell liquid (CNSL), which contains phenolic compounds with great biological potential, such as in treatments for asthenia, respiratory problems, genital infections, and skin diseases [9–12] and for use in larvicidal compounds [11, 13–16].

Larvicide activities against *Ae*. *aegypti* of technical CNSL, its main constituents cardanol and cardol, and their products of hydrogenation were evaluated. Structure-activity relationship studies revealed significant differences in larvicidal activity against *Ae*. *Aegypti* between technical CNSL and its main constituents. Technical CNSL presented an LC_50_ value of 51 ppm (μg/mL), whereas isolated cardol and cardanol showed LC_50_ values of 14.2 and 32.9 ppm, respectively. Therefore, we show that cardol is the constituent primarily responsible for the activity demonstrated by technical CNSL [13, 16].

Although the phenolic lipids isolated from CNSL show larvicide activity, these compounds do not dissolve in water. To solve this problem, Farias et al. [17] used sodium anacardate, isolated from natural CNSL, against *Ae*. *aegypti* larvae, obtaining an LC_50_ of 55.47 ppm. Mukohopadhyay et al. [18] tested cardanol emulsified in liquid vegetable soap against larvae of the same mosquito and calculated an LC_50_ of 12 ppm and an LC_50_ of 38 ppm for larvae of the *Anopheles subpictus*, while Raraswati et al. [19] studied the larvicide effect of an emulsion of natural CNSL with an extract of nut fruit soap (*Sapindus rarak* DC) against *Ae*. *aegypti* larvae in the 3rd instar stage and found an LC_50_ of 14.12 ppm.

In preliminary studies, Galdino; Beatriz [14] and Beatriz et al. [15] devised saponification reactions with these natural products to obtain water-soluble salts, aiming to develop a mixture of surfactants from CNSL and castor oil. The castor oil was employed together with CNSL as a vehicle to boost the surfactant effect of the mixture. Subsequently, the larvicide tests against *Ae*. *aegypti* showed that all compounds were active. The product termed Tensoativo do Líquido da Casca da Castanha do Caju (TaLCC-20) (CNSL:Castor oil 20:80 w/w) exhibited more effective activity at a concentration of 0.2 ppm, killing 97% of larvae within the first 24 hours, while the surfactant from only castor oil showed the lowest activity. That mixture presented efficient larvicide action and resulted in the registration of a patent [15]. Considering these results, the present study evaluated the effects of TaLCC-20 on the mortality of larvae of *Ae*. *aegypti*, Rockefeller lineage (*Ae*-Rockefeller), and on the reproductive performance, embryo development and genetic stability of Swiss mice.

## Materials and methods

### Extraction of cashew nut shell liquid

Cashew nut shell (CNS) was donated by Kardol Indústria Química in 2014. The plant material was verified by Msc. Juliana Miron Vani, and a voucher specimen was deposited (No. 51838) in the herbarium of the Federal University of Mato Grosso do Sul (UFMS). The extraction of natural CNSL from CNS was performed using the procedures of Gandhi et al. [20]. First, 250 mL of 95% ethanol was added to the round bottom flask of a Soxhlet apparatus. Then, 30 g crushed CNS was Soxhlet extracted for 6 h, after which the solvent in the thimble of the Soxhlet apparatus was colorless. Finally, the solvent was recovered from a simple distillation method. The natural CNSL was obtained as reddish brown phenolic oil (40% yields). The HPLC analysis was performed using a Shimadzu LC-6AD apparatus with a Diode Array Detector (SPD-M10Avp, Shimadzu). The analytical column was a Phenomenex Luna C18 column (4.6 × 250 mm, 5 μm). The mobile phase was methanol/water (95:5) at a flow rate of 1.5 mL/min. ^1^H-NMR spectra were recorded in CDCl_3_ (Tedia Brazil, Brazil) solution on a Bruker DPX300 spectrometer (300 MHz), and spectra was referenced to TMS using residual solvent signals as secondary standards. The ethanol employed for extraction of CNSL was AR grade (Vetec, Brazil), and the methanol used in HPLC was HPLC grade (Mallinckrodt, USA). HPLC-grade water (18 mW) was prepared using a Milli-Q system (Millipore). Solvents were filtered with spare membrane filters (0.2 μm, sterile).

### Preparation of the sodium surfactant from a mixture of cashew nutshell liquid and castor bean oil (TaLCC-20)

For these procedures, the following reagents were used: absolute ethanol VTEC, p.a., ACS reagent, 99% purity; castor bean oil EXP type 01, batch M-05-04 Celtic; sodium hydroxide Dinâmica, p.a., ACS reagent, 97% purity; distilled water; and natural CNSL obtained via Soxhlet extraction. The common procedures for producing soaps from vegetable oils were used [15]. Approximately 33.0 mL of an aqueous solution of sodium hydroxide (12.6 g) was slowly added to an ethanolic solution of 20 g natural CNSL and 80 g castor bean oil (66.00 mL ethanol) while stirring. The resulting solution was stirred for 15 minutes. After this period, the reaction medium was protected from light until complete solidification.

### Establishment and maintenance of *Ae*. *aegypti* Rockefeller colony

The prime matrices of *Ae*. *aegypti* eggs (Rockefeller line) were supplied by the Animal Biology Department, UNICAMP, Campinas-SP and Control of Endemia Superintendency (SUCEN), Marilia-SP. The establishment and maintenance of the *Ae*. *aegypti* colony and the production of eggs were realized in a creation room of the LIVe laboratory (LIVe, Laboratory of Vector Insects–Biological Science University–FCBA, Federal University of Grande Dourados) at a controlled temperature of ± 28°C, relative humidity of ± 60% and a programed photoperiod of 10 hours of dark and 14 hours of light.

For biological assay execution, *Ae*. *aegypti* eggs were supplied by LIVe.

### Larvicide assay

The larvicide bioassay was carried out with 3^rd^-stage *Ae*. *aegypti* larvae of the Rockefeller lineage (*Ae*-Rockefeller). First, eggs (on filter paper) were placed in plastic trays containing a volume of distilled water greater than 1 mL *per* larva and macerated fish feed for larval hatching (Alcon Basic® Lot 162). After five days, the larvae reached the 3^rd^ developmental stage. With a polyethylene Pasteur pipette, 20 *Ae*-Rockefeller larvae were transferred to a 50-mL beaker containing 20 mL of solution and 20 larvae. Four-hundred larvae divided into four replicates were subjected to each treatment: a negative control containing only distilled water, a positive control containing temephos at a concentration of 0.012 mg/L, and three concentrations of TaLCC-20 (0.05 mg/L, 0.5 mg/L, and 5 mg/L).

### Residual effect test

The residual effect of TaLCC was assessed for the larvicide concentration (5 ppm—5 mg/L) and for a 10× higher dose (50 ppm—50 mg/kg). Drinking water was used for the control group, which was the TaLCC dilution vehicle.

The tests were performed in triplicate. Beakers were used with 30 mL of each solution containing 20 *Ae*. *aegypti* larvae of the Rockefeller line (Ae-Rockefeller) in the 3^rd^ stage.

Larval mortality was assessed every 24 hours of exposure. Daily larval counting and replenishment continued until complete loss of the larvicide effect in the solution.

### Preclinical trial

#### Experimental animals

Swiss mice (*Mus musculus*) (30 females and 15 males) of reproductive age (8–10 weeks) with an average weight of 30 g, obtained from the State Bureau of Animal and Plant Health Protection (Agência Estadual de Defesa Sanitária Animal e Vegetal—IAGRO), were used. This study was approved by the Ethics Committee for Animal Experimentation of the Federal University of Mato Grosso do Sul (No. 401/2012).

The animals were maintained in propylene boxes; males were housed in insolation, and females were housed in pairs. The mice were allowed an adaptation period of seven days. The housing environment was an ALESCO® ventilated cabinet that was light and temperature controlled, with a photoperiod of 12 hours (12 hours of light: 12 hours of dark) and a temperature of 22 ± 2°C. The mice were provided with commercial feed (Nuvital ®) and filtered water.

Overnight mating was performed at a ratio of 1 male: 2 females, and detection of pregnancy was based on vaginal plug formation (considered day zero of gestation) [21–25].

After vaginal plug formation, health and well-being of the animals were evaluated daily. The following clinical signs of toxicity were observed: mucosal dryness, walking alterations (locomotor hypo-activity and hyperactivity), behavioral changes, diarrhea, decreased food and water intake, eye opacity, hair bristling, tremors, and morbidity [26–28].

### Experimental design

**Fig 1:**
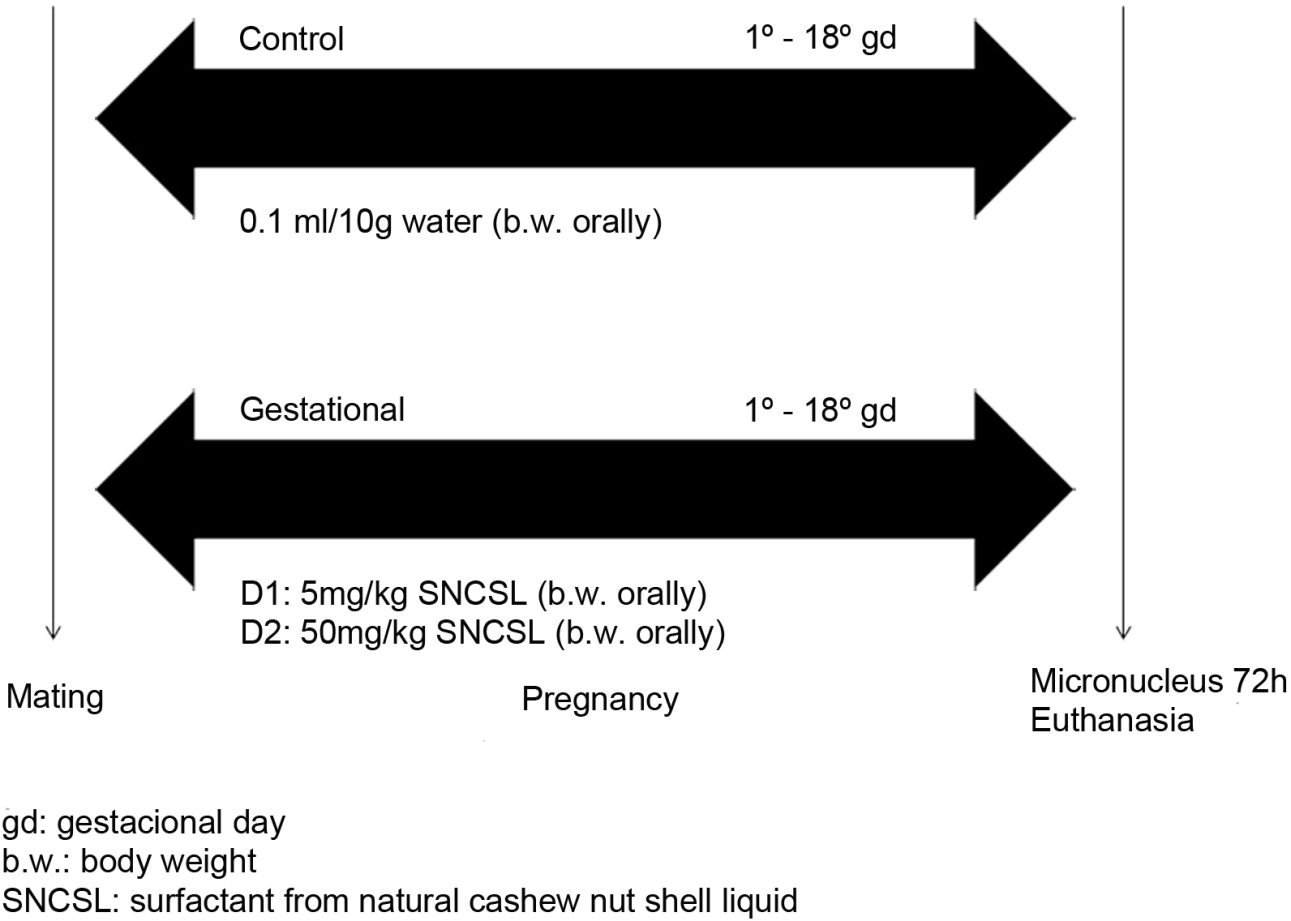
Treatment period and experimental design.

The pregnant females were divided into three groups (n = 10): control group animals received drinking water at 0.1 mL/10 g body weight (b.w.) orally (gavage) on each day of pregnancy (1^st^ to 18^th^); gestational group animals received TaLCC-20 at doses of 5 mg/kg (Gest. D1) and 50 mg/kg (Gest. D2) b.w. orally on all days of gestation (Fig 1).

The dose of 5 mg/kg (p.c., v.o.) was based on the larvicide dose, and the security dose was defined as 10× greater than the indicated for guidelines, i.e., 50 mg/kg (p.c., v.o.) [29,30].

### Biological assays

#### Reproductive performance and embryonic and fetal development (teratogenicity)

On the 18^th^ day of gestation, the animals were euthanized, followed by laparotomy, hysterectomy and oophorectomy. Analgesia was not used to euthanize mice, as analgesia could affect the frequency of damaged cells in the micronucleus assay according to studies of Hoerauf et al. [31], Heine et al. [32], Kotani et al. [33] and Souza [34]. Thus, the pregnant female mice were euthanized by cervical dislocation.

The fetuses were euthanized using isoflurane, as only the tissues and bones needed to be preserved for subsequent analysis. The micronucleus assay was not performed for the fetuses.

The spleen, heart, liver, lungs and kidneys were collected and weighed, and the fetuses and placentas were also weighed. An external systematic analysis of the fetuses was performed to detect possible external malformations, and the sex of the fetuses was determined. The number of implantations, resorptions, and live and dead fetuses was recorded. Based on these data, fetal viability (number of live fetuses/number of implantations x 100), the post-implantation loss rate (number of implantations–number of live fetuses x 100/number of implantations), the resorption rate (number of resorptions x 100/number of implantations), the placental index (placental weight/fetal weight) and the sex ratio (number of male fetuses/number of female fetuses) were obtained [21, 23–25]. Then, the suitability of the observed fetal weight for the gestational age was determined according to Oliveira et al. [21], and the fetuses were classified as follows: fetuses with an appropriate weight for their gestational age (AWGA), with a body weight within the mean weight of the control group of fetuses plus or minus the standard deviation; fetuses with a low weight for their gestational age (LWGA), with a body weight lower than the mean weight of the control group fetuses minus the standard deviation of the same group; or fetuses overweight for their gestational age (OVGA), with a body weight higher than the mean weight of control group fetuses plus the standard deviation of the same group.

Subsequently, the fetuses were randomly divided into two subgroups. The first subgroup underwent visceral analysis, for which the fetuses were fixed in Bodian’s solution (distilled water (142 mL), acetic acid (50 mL), formaldehyde (50 mL) and 95% ethanol (758 mL)) for at least seven days. Visceral analysis was performed via microdissection with strategic cuts to examine the chest and abdomen, according to Barrow and Taylor [35], and to examine the head, according to Wilson [36], as modified by Oliveira et al. [21]. Visceral changes were described based on the studies by Taylor [37], Manson and Kang [38], Damasceno et al. [26] and Oliveira et al. [21]. The second subgroup of fetuses was intended for skeletal analysis using the alizarin red technique proposed by Staples and Schnell [39], as modified by Oliveira et al. [21]. The fetuses were fixed in acetone for at least seven days. For the diaphonization process, the fetuses were eviscerated and placed in a solution of KOH (0.8%). Then, four drops of alizarin were added. This solution was replaced every 24 hours over four days. After this period, the KOH solution was discarded, and the fetuses were placed in a bleaching solution (1 L glycerin:1 L of ethyl alcohol: 0.5 L of benzyl alcohol), which was replaced every 24 hours for seven days. Skeletal changes were classified according to Taylor [37], Manson and Kang [38], Damasceno et al. [26] and Oliveira et al. [21].

All analyses were performed under a stereomicroscope (NIKON SMZ745T).

### Micronucleus assay

The technique used for the micronucleus assay was based on Hayashi et al. [40], as modified by Oliveira et al. [21]. A total of 20 μL peripheral blood was collected via tail vein puncture, deposited on a slide that was previously stained with acridine orange (1 mg/mL) and then covered with a coverslip. Samples were collected on the 18^th^ gestational day (i.e., at the end of the experiment) to assess whether TaLCC-20 had the ability to cause cumulative damage. The slides were stored in a freezer at -20°C for at least 15 days. A total of 2,000 cells/animal were analyzed under an epifluorescence microscope (Motic®; Model BA 410) at a magnification of 400×).

### Statistical analysis

The data are presented as the mean ± standard error of the mean (SEM) and were evaluated according to the nature of their distribution (parametric: ANOVA/Tukey test; nonparametric: Kruskal-Wallis/Dunn test). The chi-square test was used to compare frequencies (percents) between the control and experimental groups. The level of significance was set at p<0.05.

## Results

**Fig 2:**
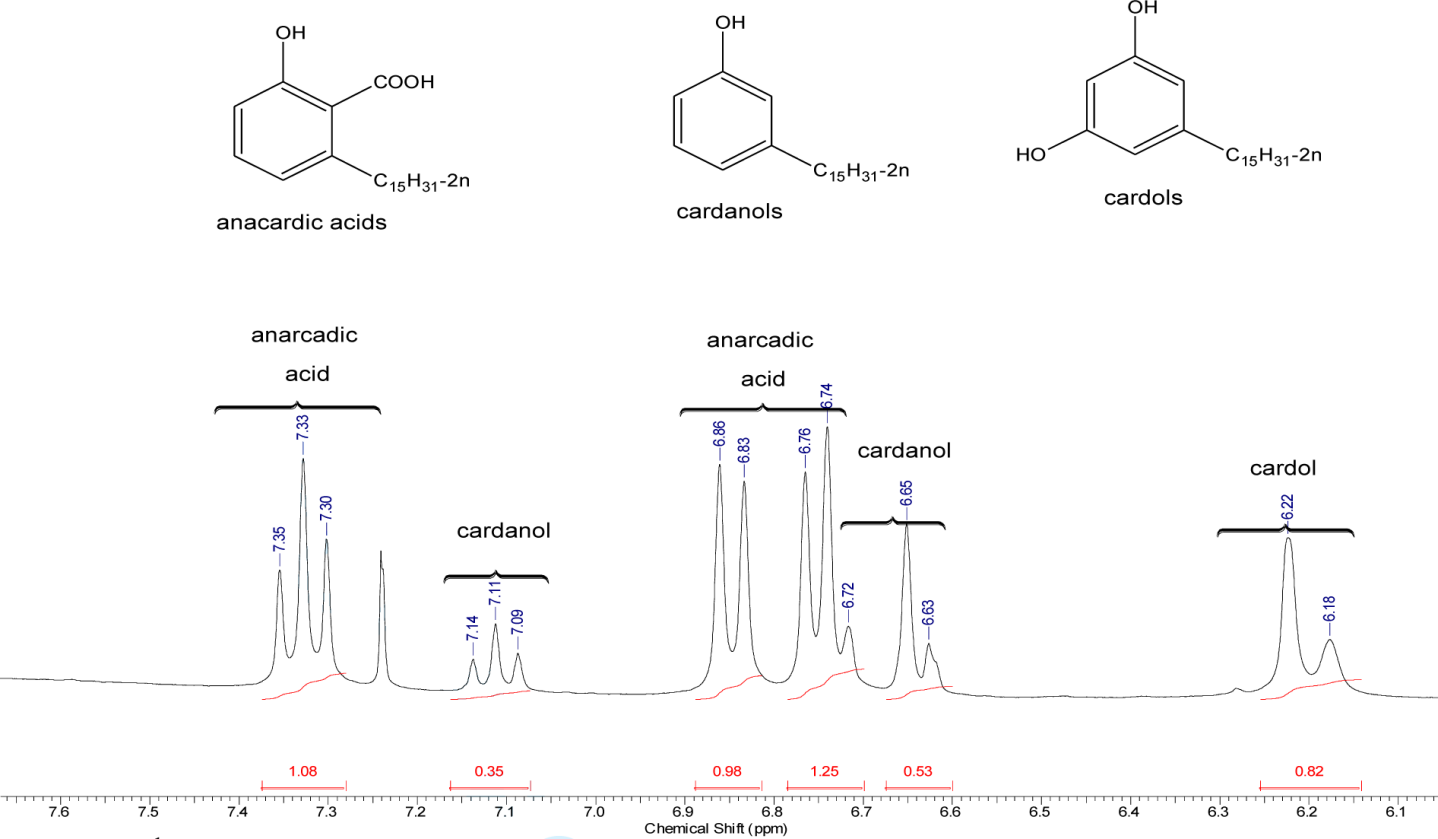
^1^H-NMR spectra of natural CNSL (expanded region for aromatic protons).

The natural CNSL used in this analysis is predominately composed of anacardic acid, as verified in the ^1^H NMR spectrum (Fig 2). The spectrum shows signals corresponding to aromatic, olefinic, methylenic, and methyl hydrogens, in agreement with reports in the literature [41]. Signals corresponding to methylcardol are not shown in the spectrum. Aromatic hydrogens are observed from 6.10–7.35 ppm. The signals between 4.95–5.87 ppm are attributed to olefinic hydrogens. The signals between 1.15–2.99 ppm are assigned to methylene hydrogens. The three aromatic hydrogens of anacardic acid are a doublet of doublets at 7.33 ppm (J = 9 and 6 Hz), integrated for 1H, and, two doublets at 6.85 ppm (J = 9 Hz) and 6.74 (J = 6 Hz), integrated for 1H each. Aromatic hydrogens of cardol are observed at 6.15–6.25 ppm, and those for cardanol are observed at 6.60–6.75 ppm together with a doublet of doublets at 7.11 ppm. In accordance with the integration of the signals for aromatic hydrogens, our findings suggest that the ratio of anacardic acid/cardanol/cardol is 4:1.36:1, i.e., the approximate composition of natural CNSL is 62.3% anacardic acid, 21.4% cardanol and 15.7% cardol, shown in the expanded view of the aromatic region in Fig 2.

**Fig 3:**
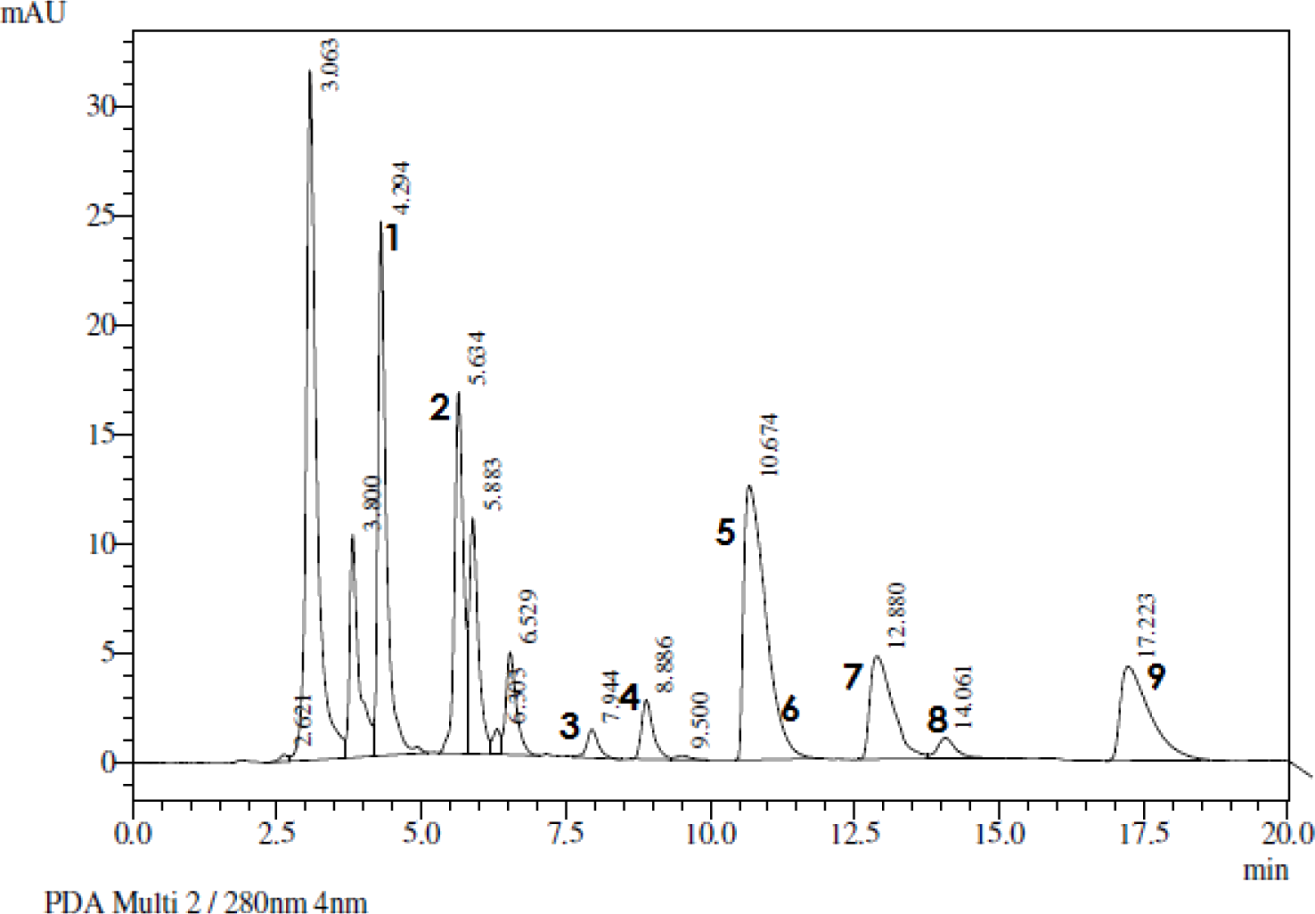
HPLC chromatogram obtained from natural CNLS, cardol (1, 2 and 3), cardanol (4, 6 and 8), and anacardic acid (6, 7 and 9).

Shows the HPLC chromatogram obtained for natural CNSL. Peaks 1, 2 and 3 correspond to monoene-, diene- and triene-cardol, peaks 4, 6 and 8 correspond to monoene-, diene- and triene-cardanol, and peaks 5, 7 and 9 correspond to monoene-, diene- and triene anacardic acid (Fig 3) [42,43].

### CNSL evaluation

In this study, we developed a mixture of surfactants with natural CNSL and sodium ricinoleate. Common procedures for producing surfactants from vegetable oils were used [15]. The substrates were reacted with sodium hydroxide using pure castor bean oil in the presence of the newly extracted CNSL, resulting in a mixture of anionic surfactants containing sodium ricinoleate (largely originating from the triglyceride of ricinoleic acid) and sodium anacardate and phenolates (originating from CNSL).

This surfactant was prepared via a saponification reaction with NaOH solidified after 10 days at room temperature protected from light.

### Evaluation of larvicide potential

**Fig 4:**
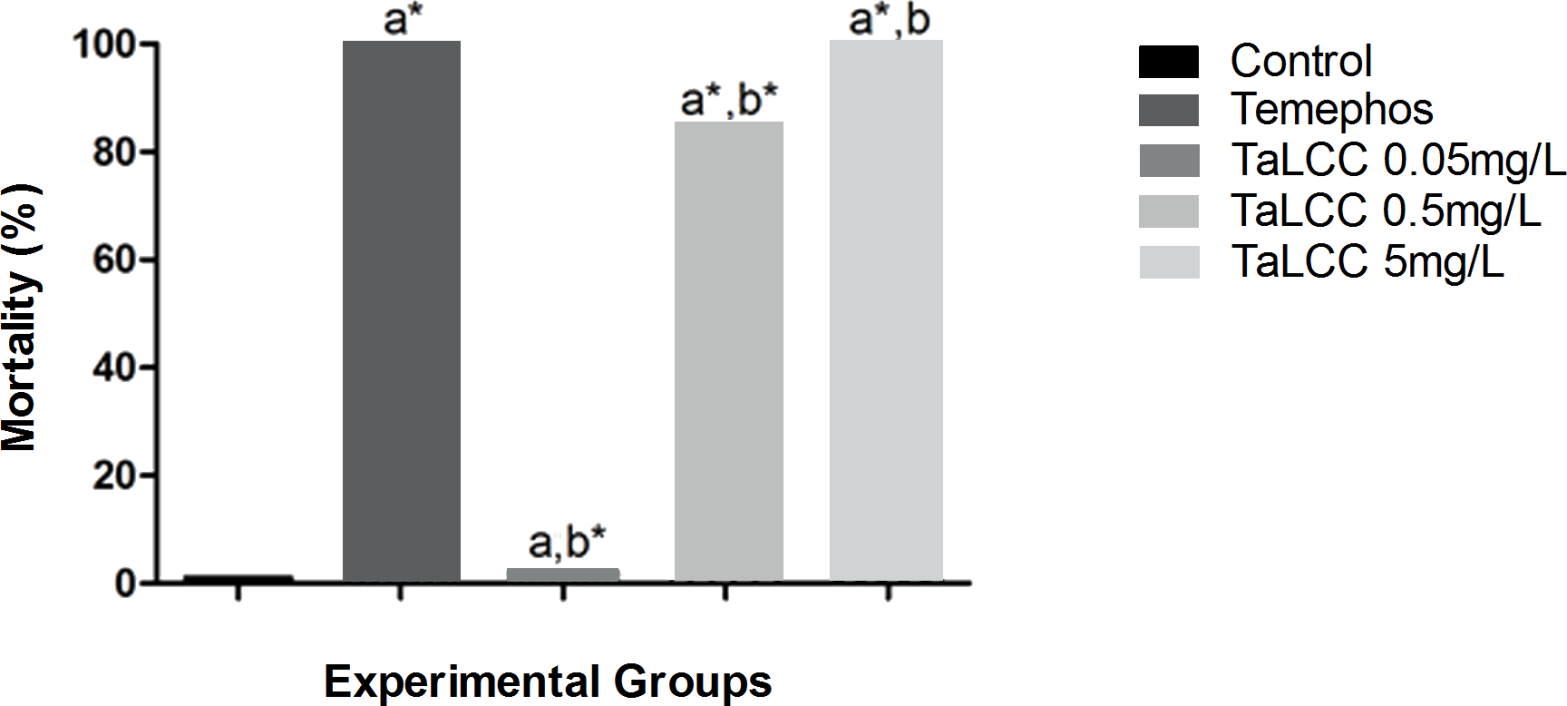
Mortality of larvae at the end of treatment exposure. Treatment exposures were statistically compared to the negative control (a) and the positive control (b). * Significant difference (chi-square test, p <0.05).

Temephos causes 100% death of larvae within three hours (p <0.05). This result is considered the gold standard and was therefore used as a positive control (Table 1). TaLCC-20 exhibited larvicidal potential compared with the negative control, as there was a decrease (p<0.05) in larval viability observed at all the tested concentrations. Compared with the positive control, the two lowest concentrations exhibited lower larvicidal activity (p<0.05), while the highest concentration presented the same effectiveness as temephos (i.e., causing death of the larvae at the same frequency over the same time interval) (Fig 4). These results demonstrate that temephos and TaLCC-20, administered at a dose of 5 mg/L, result in the same gold-standard response.

**Table 1:** Percentage of dead larvae at different exposure times to treatment.

Treatments					
Exposure Time	Control	Temephos	TaLCC 0.05 mg/L	TaLCC 0.5 mg/L	TaLCC 5 mg/L
Mortality (%)					
3 hours	1.25	100	0	1.25	100
6 hours	0	0	0	7.5	0
9 hours	0	0	0	10	0
12 hours	0	0	0	18.75	0
24 hours	0	0	0	16.25	0
48 hours	0	0	2.5	18.75	0
72 hours	0	0	0	12.5	0

### Residual effects

**Fig 5:**
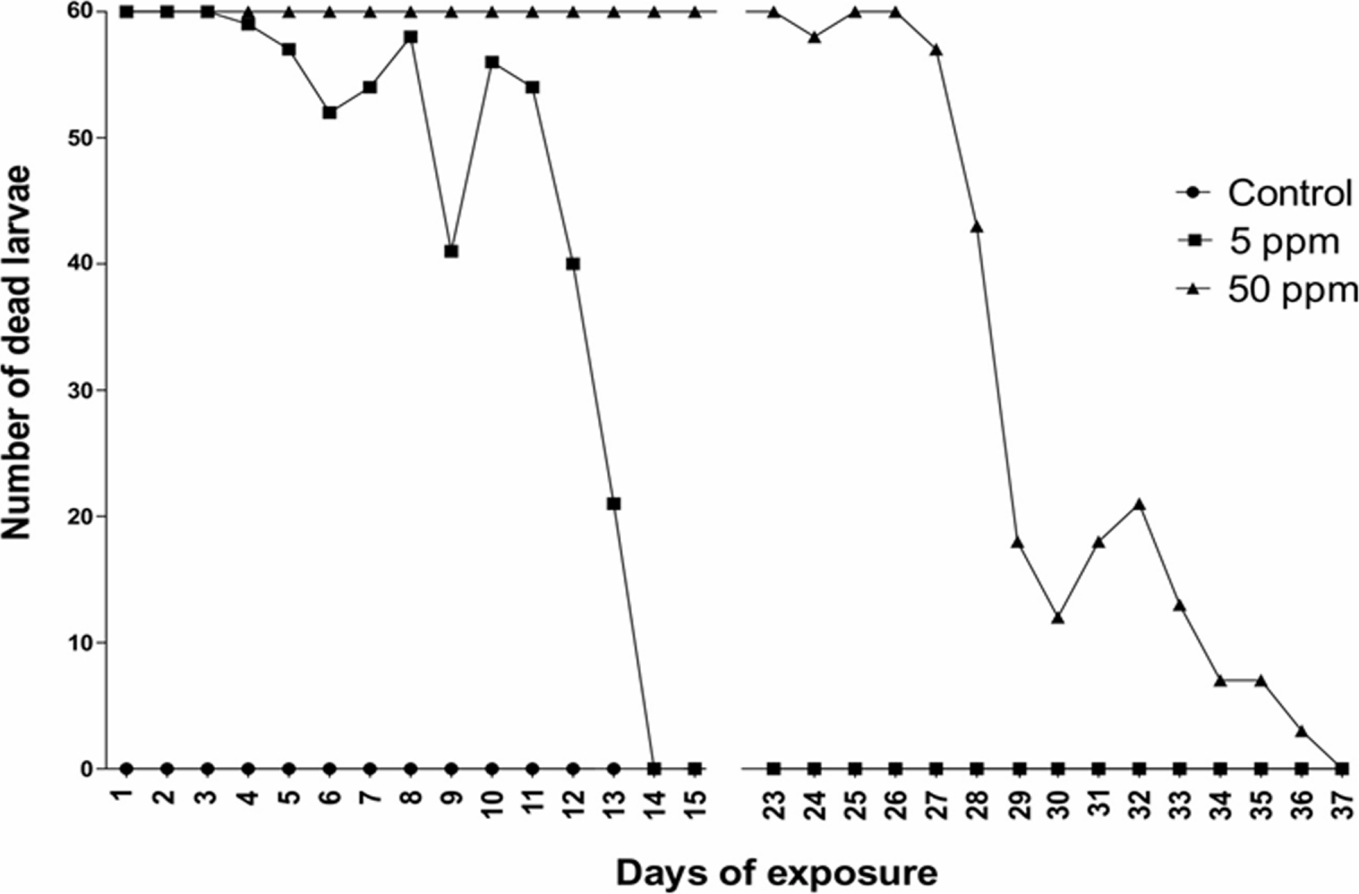
Residual effect of the mixture of cashew nut shell liquid and castor oil on 3^rd^ instar larvae of *Aedes aegypti*.

The control group did not exhibit larvae lethality throughout the experiment. At the 5 ppm (5 mg/kg) concentration, 100% of larval mortality was observed through the 3^rd^ day of exposure. After the 4^th^ day, a decreased larvicidal effect was observed, which was completely lost by the 13^th^ day. The concentration 10× higher than the larvicide dose (50 ppm-50 mg/kg) caused 100% mortality of the larvae through the 22^nd^ day of exposure. After this time, larval mortality decreased until no morality was noted on the 36^th^ day (Fig 5).

### Evaluation of reproductive toxicology

#### Evaluation of biometric parameters

Despite the random distribution of animals in the experimental groups, the animals of the group control corresponded to higher initial weights than animals of the experimental groups. This weight difference was maintained until the end of pregnancy. However, the weight gain and net weight gain were similar among all groups (p>0.05). A reduction in uterus weight (p<0.05) was observed in the Gest. D2 group compared with the control group (Table 2).

**Table 2:** Parameters related to growth development and organ weight of the females treated with TaLCC.

Biometrics Parameters					
Experimental Groups	Initial Weight	Final Weight	Weight Gain	Weight Utero	Liquid Weight Gain
Control	35.8±1.45^b^	61.01±1.88^b^	25.21±1.24^a^	20.48±0.99^b^	4.73±1.12^a^
Gest. D1	27.8±1.32^a^	52.46±2.46^a, b^	24.66±3.20^a^	19.48±0.50^a, b^	5.25±2.84^a^
Gest. D2	31.4±1.15^a, b^	49.00±2.61^a^	17.60±2.70^a^	15.94±1.70^a^	1.66±1.75^a^
**Absolute Weight Organs (g)**					
	**Heart**	**Lung**	**Spleen**	**Kidney**	**Liver**
Control	0.19±0.011^a^	0.23±0.01^a^	0.17±0.02^a^	0.42±0.01^b^	2.77±0.06^b^
Gest. D1	0.17±0.011^a^	0.36±0.03^b^	0.15±0.01^a^	0.24±0.02^a^	0.60±0.29^a^
Gest. D2	0.16±0.008^a^	0.19±0.02^a^	0.16±0.02^a^	0.35±0.01^b^	2.13±0.09^b^
**Relative Weight Organs (g)**					
	**Heart**	**Lung**	**Spleen**	**Kidney**	**Liver**
Control	0.003±0.0002^a^	0.004±0.0002^a^	0.003±0.0006^a^	0.007±0.0002^b^	0.04±0.002^b^
Gest. D1	0.003±0.0002^a^	0.007±0.0006^b^	0.003±0.0006^a^	0.005±0.0004^a^	0.01±0.005^a^
Gest. D2	0.003±0.0002^a^	0.004±0.0006^a^	0.003±0.0002^a^	0.007±0.0004^b^	0.04±0.001^b^

Different letters (a and b) indicate statistically significant differences: p<0.05 (Test a: Analysis of Variance/Tukey; Test b: Kruskal-Wallis/Dunn).

Regarding the relative weights of organs, an increased weight (p<0.05) was observed for the lungs and a decreased weight (p<0.05) for the kidneys and the liver in the Gest. D1 group compared with the control group (Table 2).

### Evaluation of reproductive performance

The number of implants, live and dead fetuses, mean number of fetuses, resorption rate, placental weight and sex ratio did not differ (p>0.05) among the experimental groups. However, there were decreases (p<0.05) in the fetal viability rate, post-implantation losses and fetal weight and increases (p<0.05) in the number of resorptions and placental index. Despite these differences, the weight of the fetuses in the experimental groups was considered suitable for the gestational age (Table 3).

**Table 3:** Reproductive parameters for females treated with TaLCC.

Experimental Groups			
Parameter	Control	Gest. D1	Gest. D2
Implants	14.00±0.60^a^	12.80±0.42^a^	13.70±0.67^a^
Live Fetuses	13.50±0.70^a^	12.60±0.30^a^	11.10±1.34^a^
Dead Fetuses	0.00±0.00^a^	0.10±0.10^a^	0.30±0.21^a^
Average Number Fetuses	13.4±0.72^a^	12.6±0.30^a^	11.1±1.34^a^
VF	96.46±3.20^b^	98.67±0.89^b^	79.58±7.77^a^
TPPI	82.46±3.27^a,b^	85.87±1.27^b^	65.88±7.51^a^
Reabsorption	0.70±0.40^a^	0.10±0.10^a^	2.30±0.97^b^
TR	5.21±2.93ª^,b^	0.67±0.67^a^	18.36±8.10^b^
PP (g)	0.09±0.002ª	0.09±0.002^b^	0.08±0.002^a^
IP	0.07±0.002^a^	0.07±0.001ª	0,08±0,002^b^
PF (g)	1.21±0.01^b^	1.24±0.01^b^	1.09±0.01^a^
APIP		PAIP	PAIP
RS	0.94±0.20^a^	1.31±0.45^a^	1.01±0.16^a^

Different letters (a and b) indicate statistically significant differences: p<0.05 (Test a: Analysis of Variance/Tukey; Test b: Kruskal-Wallis/Dunn). Fetal viability; TPPI: rate of post-implantation losses; TR: resorption rate; PP: placental weight; PF: fetal weight; IP: placental index; APIP: adequacy of weight to age of pregnancy; PAIP: proper weight to age of pregnancy; RS: sex ratio.

### Evaluation of embryonic and fetal development: external, visceral and skeletal malformations

Malformations of the external limbs and tail were observed in all experimental groups, though at a low frequency that did not significantly differ among groups (Table 4).

**Table 4:** Relationship and frequency of external malformations in the offspring of females treated with TaLCC.

Experimental Groups			
Parameters	Control	Gest.D1	Gest.D2
**Members**			
Analyzed Fetuses	134	126	111
Normal Fetuses	125	121	109
Retr.Post.Unilateral	5	5	2
Retr.Pos.Bilateral	2	0	0
Retr.Ant.Unilateral	1	0	0
Phocomelia	1	0	0
Freq.Malf.	**9**	**5**	**2**
%M.F.	**6.2**	**3.97**	**1.80**
**Tail**			
Normal Fetuses	132	124	108
Rolled up tail	2	2	3
Freq.Malf.	**2**	**2**	**3**
%M.F.	**1.49**	**1.59**	**2.78**
**Nose**			
Normal Fetuses	134	126	110
Hematoma	0	0	1
Freq.Malf.	**0**	**0**	**1**
%M.F.	**0**	**0**	**0.91**

Freq.Malf.: frequency of malformations; %M.F.: average value percentage of malformation; Retr.: retroversion; Ant.: anterior; Post.: posterior. Statistically compared with the control (chi-square test, p >0.05).

Visceral observed malformations included hydrocephaly and hydronephrosis. These conditions occurred at the same frequency in all experimental groups (Table 5).

**Table 5:** Relationship and frequency of visceral malformations in the offspring of females treated with TaLCC.

Experimental Groups			
Parameters	Control	Gest. D1	Gest. D2
**Cerebro-Hydrocephalus**			
Analyzed Fetuses	67	63	56
Normal Fetuses	20	26	18
Hidro.Light	44	33	38
Hidro.Severe	0	4	0
Freq.Malf.	**44**	**37**	**38**
%M.F.	**65.67**	**58.73**	**67.86**
**Region Urogenital—Hydronephrosis**			
Normal Fetuses	64	60	52
Hidro.Light	3	3	4
Freq.Malf.	**3**	**3**	**4**
%M.F.	**4.48**	**4.76**	**7.14**

Freq.Malf.: frequency of malformations; %M.F.: average-value percentage of malformation; Hidro.: hydronephrosis; Hidro.: hydrocephalus. Statistically compared with the control (chi-square test, p >0.05).

Regarding the detected skeletal malformations, an absence of or reduced ossification was observed in the phalanges, metacarpals, metatarsals, sternal centers, palate, presphenoid, parietal and ribs. The occurrence of these malformations was similar in all experimental groups. However, there was an increase (p<0.05) in the degree of ossification reduction recorded in the palate and presphenoid in the TaLCC-20-treated groups compared with the control group (Table 6).

**Table 6:** Relationship and frequency of skeletal malformations in the offspring of females treated with TaLCC.

Experimental Groups				
Parameters		Control	Gest.D1	Gest. D2
**Members**				
Analyzed Fetuses		67	63	55
Normal Fetuses		0	2	0
Phalanges.	Absente	60	52	51
	O.R.	1	0	4
Metac.Metat.	Absent	6	7	0
	O.R.	0	2	0
Freq.Malf.		**67**	**61**	**55**
%M.F.		**100**	**92.82**	**100**
**Sternum**				
Normal Fetuses		56	43	45
Sternal centers	Absent	3	2	3
	O.R.	8	18	7
Freq.Malf.		**11**	**20**	**10**
%M.F.		**16.41**	**31.75**	**18.18**
**Head and Jaw**				
Normal Fetuses		57	13	37
Pal.Sph.	Absent	0	0	0
	O.R.	10	50	17
Parietal	Absent	0	0	1
Freq.Malf.		**10**	**50**	**18**
%M.F.		**14.92**	79.36*	32.73*
**Column**				
Normal Fetuses		66	63	55
Rib Agenesis		1	0	0
Freq. Malf.		**1**	**0**	**0**
%M.F.		**1.49**	**0**	**0**

Freq.Malf.: frequency of malformations; %M.F.: average-value percentage of malformation; Metac.: metacarpus; Metat.: metatarsal;; Pal: palate Presf.: sphenoid O.R.: reduced ossification.

* Statistically significant difference compared with the control (chi-square test, p <0.05)

### Toxicogenic evaluation: micronucleus assay

**Fig 6:**
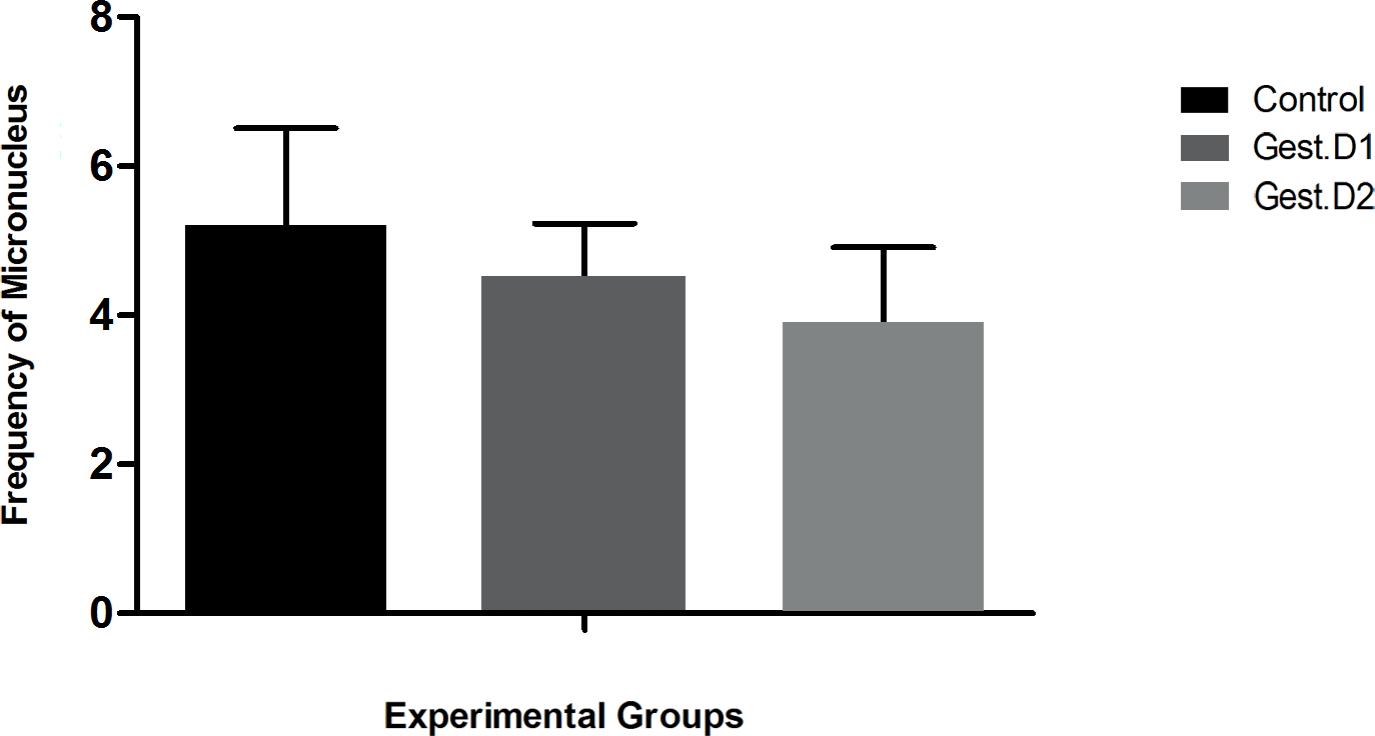
Micronucleus frequency after treatment. (Test: analysis of variance/Tukey, p<0.05).

TaLCC-20 showed no mutagenic activity (Fig 6), as there was no difference (p<0.05) in the frequency of micronuclei between the various experimental groups. The mean frequency of micronuclei was 5.20 ± 1.31 in the control group and 4.50 ± 0.73 and 3.89 ± 1.02 in the Gest. D1 and Gest. D2 groups, respectively, on the 18^th^ gestational day (after treatment throughout the gestational period), demonstrating that TaLCC-20 does not cause cumulative genetic damage.

## Discussion

Larvicides are generally used in fumigation and/or deposited in water tanks (regardless of whether they contain drinkable water) [44]. Thus, important concerns regarding the use of larvicides include their inhalation, accidental poisoning and contamination of drinking water consumed by the population, especially for cooking and hydration.

According to Machado et al. [7], the best results recorded to date in terms of preventing an increase in the number of dengue cases in Brazil have been obtained using diflubenzuron and, more recently, the juvenile hormone analogue pyriproxifen, which is indicated and distributed by the Brazilian Ministry of Health [45,46]. Thus, these two commercial products have largely been used to restrain the reproduction of the vector mosquitoes responsible not only for dengue but also for chikungunya fever and Zika viruses, which are re-emerging in Brazil and other parts of the world [47,48].

It is well established in the literature that the use of pesticides, insecticides, larvicides and/or growth inhibitors is directly linked to genetic/genomic instability, which can increase predisposition to cancer [8,49–55], in addition to altering reproductive performance and causing hormonal disorders and male infertility [56,57].

According to Barros et al. [57], the aforementioned effects may be caused by diflubenzuron, which has been widely used in the recent epidemics and is efficient in combating mosquitoes/vectors but toxic. No information on pyriproxifen is available in the literature regarding its mutagenic and/or teratogenic effects. In the absence of such data, pyriproxifen must be used with caution and considered potentially toxic, calling for the need of biomonitoring. Given these concerns, the development of less toxic products with available safety information regarding their use is urgently needed.

For a suitable product to be developed, the product must exhibit equal or better efficiency than commercially available products in combating larvae and/or mosquitoes/vectors in other life stages and be more selective (i.e., be able to control vector survival and reproduction without causing genetic and/or reproductive toxicity to animals or humans).

Developing such a product would solve important public health issues without causing harm to the population that could potentially require further investments to treat chronic non-degenerative diseases, such as cancer, subfertility and infertility.

CNSL exhibits larvicidal activity, as previously described [11,13,16]. In addition, preliminary data from Galdino and Beatriz [14] and Beatriz et al. [15] demonstrated that mixtures of sodium surfactants from natural CNSL and castor bean oil exhibit larvicidal activity; among the various tests that were performed, the best results were observed at a ratio of 20:80. Thus, the same mass/mass relationship was chosen for this pilot study. We present a larvicide with natural peculiarities in its composition that was obtained from a combination of natural CNSL and castor bean oil and takes the form of a sodium surfactant. This formulation increases the solubility of CNSL, allowing further dispersion of the product at mosquito/vector breeding sites, offering further larvicidal potential.

New tests performed with TaLCC have demonstrated its ability to kill 100% of *Ae*. *aegypti* larvae within a mean time of three hours at a dose of 5 mg/L (ppm). These results are considered by the Ministry of Health to represent the gold standard for commercial compounds, such as temephos, which also induces the death of 100% of larvae within a few hours of exposure [58,59]. In addition to achieving the recommended gold standard, our results suggest that the TaLCC-20 product has no side effects in mammals, including humans. These findings confirm the better cost/benefit ratio of TaLCC-20 compared, for example, with temephos, because although the latter product shows high efficiency, it is toxic to both the environment and humans [51].

The residual effect of TaLCC for maintaining lethality of 100% of larvae lasted for up to 13 days and 22 days at the used doses of 5 and 50 ppm, respectively. After these periods, larvicide action was gradually lost and ceased within 13 and 37 days for the doses of 5 and 50 ppm, respectively. These results demonstrate that a formulation of TaLCC not only improves the solubility of other products prepared based on cashew nuts but also improves the time of action. According to Guissoni et al. [11], TaLCC can kill larvae within 6 days (100% death) with a residual effect remaining for 14 days. Therefore, the TaLCC has a greater residual effect, and this period is increased by 23 days, which allows for greater action over time and is a highly desired feature of larvicides

In addition to these benefits, TaLCC-20 presents strong commercial appeal because it is easily produced, exhibits good yield and is inexpensive. Thus, TaLCC-20 may provide and inexpensive solution for a public health issue that is associated with high costs in various regions of the world. In addition, this product can prevent many deaths and improve the quality of life of millions of people who are affected by the symptoms caused by dengue, chikungunya fever and Zika virus. These infections and their complications are also responsible for the absence of workers from their workplaces, causing major harm to the production sectors of various countries [60,61].

The toxic effects of xenobiotics on genetic material and/or reproductive health can be evaluated based on mutagenesis, reproductive performance, embryonic and fetal development and teratogenesis assays [62–64]. Products that do not induce damage according to these biological assays are favored for commercialization [62]. Thus, the present study evaluated TaLCC-20 in a preclinical model to determine whether it can be safely used and to predict the health risks of exposure to this product in mammals, including humans.

The guidelines for reproductive toxicology [30] and genetic toxicology [65,66] and the National Health Surveillance Agency [67] indicate that preclinical trials should be carried out using doses intended for use in humans (or using the doses to which humans may be exposed) and another dose 10 times higher. The lower dose can only be considered safe if the higher dose is free of adverse effects. Thus, in the present study, we evaluated a dose that has larvicidal potential (5 mg/kg) and a dose 10 times higher (50 mg/kg) to assess possible maternal effects and embryonic toxicity. For a compound to be released for use and commercialization, evidence of a lack of mutagenic and teratogenic effects and the cost/benefit ratio associated with its use must be evaluated [30, 67,68].

According to Zhang et al. [69] and Sally et al. [70], the state of pregnancy modifies an individual’s metabolism, which may induce the body to become more susceptible to xenobiotic effects. Hence, also performed a mutagenesis analysis of pregnant females.

Considering the above issues, the results of the present study were promising, including 1) the high larvicidal efficiency of the product, and2) the lack of changes in embryonic and fetal development caused by the product and no detected genetic instability. Such effects can be correlated with infertility, teratogenesis/congenital malformations and cancer [8,51,53,56,57], which are important health issues that may negatively impact public health systems because, as chronic diseases, they require large investments to treat and maintain the quality of life of patients.

Regarding the mutagenic capacity of TaLCC-20, the micronucleus assay revealed no genetic damage. This result is important because some products used against mosquitoes, such as temephos and diflubenzuron, are mutagenic and/or cause changes in DNA [8,51].

The preclinical trial also indicated an absence of toxicity based on the biometric parameters that were evaluated. According to other studies, weight loss and changes in the absolute and relative weights of organs may be indicative of toxicity [71,72]. In the present study, although the animals were randomly distributed, the Gest. D1 group presented the lowest mean weight. However, the recorded weight gain and net weight gain showed no significant variation, indicating an absence of TaLCC-20 toxicity. The reduction in uterus weight recorded for the Gest. D2 group can be explained by the lower number of fetuses per litter in this group; thus, it is not indicative of toxicity. The low initial weight of the animals in the Gest. D1 group may explain the reduction in the weights of the lungs, kidneys and liver observed in this group. In general, these parameters are not considered signs of toxicity because xenobiotics causing damage to the body particularly lead to enlargement of the liver and kidneys, which are organs that are directly involved in metabolism and excretion. Furthermore, toxicity generally results in an increase in the activity of these organs, which would be consistent with their increased size.

Regarding reproductive performance and embryonic and fetal development, pregnant females may be exposed to a test compound in different stages to predict any interference with implantation (treatment from the 1^st^ to the 4^th^ gestational day), organogenesis (treatment from the 5^th^ to the 15^th^ gestational day) [23] or fetal development (treatment from the 15^th^ to 18^th^ gestational day) [73]. The literature also describes pregnancy treatments performed to assess whether a compound exhibits a cumulative effect or if it can affect more than one embryonic or fetal developmental stage [74,75]. In the present study, a gestational treatment protocol was used, and reductions in the fetal viability rate and fetal weight were observed. There were also increases in the number of resorptions and the placental index. Among these parameters, the reduction in fetal viability requires further attention.

The reduction in fetal weight and the increase in the placental index are not worrisome because, despite these differences, the fetuses continued to show an adequate weight for their gestational age. In general, a low weight of fetuses after birth is associated with an increased placental index, which is an adaptation of the maternal body in an attempt to increase the provision of nutrients to the fetus and, thus, allow proper development [76,77]. The increased number of resorptions is not a critical finding because the resorption rate, which represents complementary data, showed no difference among the experimental groups. Regarding embryonic and fetal development, there was no increase in the frequency of external or visceral malformations in relation to the control group.

The observed visceral malformations (hydrocephalus and hydronephrosis) may be normal variations, as they were also present in the control group [22,23,37]. Furthermore, the fetuses were collected prematurely, although the procedure was performed as indicated by the literature [22,37]. These studies also indicate that these changes may revert at the end of pregnancy or after birth [21,37], corroborating the notion that they are normal variants. In relation to skeletal malformations, the only significant changes observed in the treated groups were those related to reduced palate and presphenoid and parietal ossification. However, these fetuses were collected early, and thus, the ossification process was interrupted. This may explain the damages that were observed, while the ossification process can still be completed after birth. We suggest that this is not a factor that discourages the use of CNSL.

Thus, the cost-benefit relationship observed for issues related to reproductive performance and embryonic and fetal development is also positive and supports the use of TaLCC-20, especially because the available commercial products that are indicated for use by the government, such as temephos [51] and diflubenzuron [8], are reported to be toxic to reproductive health and are possible teratogens.

In summary, TaLCC-20 is considered an effective larvicide that does not induce genetic damage. In addition, the cost-benefit relationship associated with changes in reproductive performance and embryonic and fetal development appears to be positive. These findings indicate that it is an important product to be explored for use as a natural larvicide that can be employed against the mosquitoes/vectors responsible for dengue, chikungunya fever and Zika virus. These are emerging and/or re-emerging diseases that require proper management while minimizing harm to the population and the environment. Therefore, CNSL is considered an important alternative to commercial products that are toxigenic, although effective.

## References

[1] Brasil—Secretaria de Vigilância Sanitária/ Ministério da Saúde. Boletim Epidemiológico—Volume 47—n° 34–2016—Monitoramento dos casos de dengue e febre de chikungunya até a semana epidemiológica 37. 2016. Available from: htpp://http://portalsaude.saude.gov.br/images/pdf/2016/outubro/18/2016-029-Dengue-publicacao-n-34.pdf. Cited 23 October, 2016.

[2] Gatherer D, kohl A. Zika virus: a previously slow pandemic spreads rapidly through the Americas. J Gen Virol. 2015; 97:269–73. doi: 10.1099/jgv.0.000381 26684466 10.1099/jgv.0.000381

[3] Marcondes CB, Ximenes MF. Zika virus in Brazil and the danger of infestation by *Aedes* (Stegomyia) mosquitoes. Rev Soc Bras Med. 2015; 49:4–10. doi: 10.1590/0037-8682-0220-2015 26689277 10.1590/0037-8682-0220-2015

[4] Deeba F, Islam A, Kazim SN, Naqvi IH, Broor S, Ahmed A, et al Chikungunya virus: recent advances in epidemiology, host pathogen interaction & vaccine strategies. Pathog Dis. 2015; 74: pii: ftv119. doi: 10.1093/femspd/ftv119 26657109 10.1093/femspd/ftv119

[5] Madariaga M, Ticona E, Resurrecion C. Chikungunya: bending over the Americas and the rest of the world. Braz J Infect Dis. 2015; 20:91–8. doi: 10.1016/j.bjid.2015.10.004 26707971 10.1016/j.bjid.2015.10.004 PMC9425360

[6] Marano G, Pupella S, Vaglio S, Liumbruno GM, Grazzini G. Zika virus and the never-ending story of emerging pathogens and transfusion medicine. Blood Transfus. 2015; 14:95–100. doi: 10.2450/2015.0066-15 26674815 10.2450/2015.0066-15 PMC4786129

[7] Machado AAV, Estevam AO, Sales A, Brabes KCS, Croda J, Negrão FJ. Direct Costs of Dengue Hospitalization in Brazil: Public and Private Health Care Systems and Use of WHO Guidelines. PLos Negl Trop Dis. 2014; 8: e3104 doi: 10.1371/journal.pntd.0003104 25188295 10.1371/journal.pntd.0003104 PMC4154670

[8] Barros AL, Souza VV, Navarro SD, Oesterreich AS, Oliveira RJ, Kassuya CAL, et al Genotoxic and Mutagenic Effects of Diflubenzuron, an Insect Growth Regulator, on Mice. J Toxicol. Environ. Health. A. 2013; 76:1003–6. doi: 10.1080/15287394.2013.830585 24168035 10.1080/15287394.2013.830585

[9] Mazzetto SE, Lomonaco D. Óleo da castanha de caju: oportunidades e desafios no contexto e sustentabilidade industrial. Química Nova. 2009; 32:732–741. doi: 10.1590/S0100-40422009000300017

[10] Carvalho ALN, Annon R, Torres LHL, Durao ACCSD, Shimada ALB, Almeida FM, et al Anacardic Acids from Cashew Nuts Ameriolate Lung Damage Induced by Exposure to Diesel Exhaust Particles in Mice. Evid Based Complement Alternat Med. 2013; 2013: 549879 doi: 10.1155/2013/549879 23533495 10.1155/2013/549879 PMC3600199

[11] Guissoni ACP, Silva IG, Geris R, Cunha LC, Silva HHG. Atividade larvicida de *Anacardium occidentale* como alternativa ao controle de *Aedes aegypti* e sua toxicidade em *Rattus norvegicus*. Rev Bras Plantas Med. 2013; 15: 363–367. doi: 10.1590/S1516-05722013000300008

[12] Hamad FB, Mubofu EB. Potential Biological Applications of Bio-Based Anacardic Acids and Their Derivates. Int J Mol Sci. 2015; 16:8569–90. doi: 10.3390/ijms16048569 25894225 10.3390/ijms16048569 PMC4425097

[13] Lomonaco D, Santiago GMP, Ferreira YS, Arriaga AMC, Mazzeto SL, Mele G, et al Study of techical CNSL and its main componentes as new green larvicides. Green Chem. 2009; 11:31–33. doi: 10.1039/B811504D

[14] Galdino TG, Beatriz A. Síntese de sais surfactantes a partir do líquido da castanha de caju utilizados no combate à dengue. Feira Brasileira de Ciências e Engenharia. 2013; (10: 2013: São Paulo), São Paulo: EPUSP.

[15] Beatriz A, De Lima D, Souza AP, Galdino GT, Silva ECR, Ito FM, Gomes RS, Arruda EJ. Processo de produção e uso de misturas de surfactantes iônicos do líquido da casca da castanha do caju e do óleo de mamona como larvicida. 2015; Brasil. Patente: Privilégio de Inovação. Número do registro: BR10201500723. Data de depósito: 16/03/2015. Instituição de registro: INPI—Instituto Nacional da Propriedade Industrial.

[16] Torres RC, Garbo AG, Walde RZML. Characterization and bioassay for larvicidal activity of *Anacardium occidental*e (cashew) shell waste fractions against dengue vector *Aedes*. Parasitol Res. 2015; 114:3699–702. doi: 10.1007/s00436-015-4598-5 26099240 10.1007/s00436-015-4598-5

[17] Farias DF, Cavalheiro MG, Viana SM, De Lima GP, da Rocha-Bezerra LC, Ricardo NM, et al Insecticidal action of sodium anacardate from Brazilian cashew nut shell liquid against *Aedes aegypti*. 2009; J Am Mosq Cont As. 25:386–89. 10.2987/08-5851.1 19852234

[18] Mukhopadhyay AK, Hati AK, Tamizharasu W, Babu PS. Larvicidal properties of cashew nut shell liquid (Anacardium occidentale L) on immature stages of two mosquito species. J Vector Borne Dis. 2010; 47: 257–260. 21178220

[19] Raraswati GR, Sudarsono, Mulyaningsih B. Larvicidal Activity of A Mixture of Cashew Nut Shell Liquid and Water-Soluble Extract of Soap Nut Fruit (Sapindus rarak DC.) Against 3rd Instar Larvae of *Aedes aegypti*. Biology, Medicine, & Natural Product Chemistry. 2014; 3:59–64. doi: 10.14421/biomedich.2014.32.53–57

[20] Gandhi TS, Dholakiya B Z, Patel MR. Extraction protocol for isolation of CNSL by using protic and aprotic solvents from cashew nut and study of their physico-chemical parameter. Pol J Chem Tech. 2013; 15:24–7. https://doi.org/10.2478/pjct-2013-0062.

[21] Oliveira RJ, Kanno TYN, Salles MJS, Lourenão ACS, Ribeiro LR, Freiria GA, et al Effects of the polysaccharide ß-glucan on clastogenicity and teratogenicity caused by acute exposure to cyclophosphamide in mice. Regul Toxicol Pharmacol. 2009; 53:164–73. doi: 10.1016/j.yrtph.2008.12.007 19168112 10.1016/j.yrtph.2008.12.007

[22] Gonçalves CA, Siqueira JM, Carollo CA, Mauro MO, David N, Cunha-Laura A L, et al Gestational exposure to *Byrsonima verbascifolia*: Teratogenicity, mutagenicity and immunomodulation evaluation in female Swiss mice. J Ethnopharmacol. 2013; 150: 843–850. doi: 10.1016/j.jep.2013.09.012 24140582 10.1016/j.jep.2013.09.012

[23] David N de, Mauro MO, Gonçalves CA, Pesarini JR, Strapasson RLB, Kassuya CAL, et al *Gochnatia polymorpha* ssp. floccosa: Bioprospecting of an anti-inflammatory phytotherapy for use during pregnancy. J Ethnopharmacol. 2014; 154:370–9. doi: 10.1016/j.jep.2014.04.005 24727192 10.1016/j.jep.2014.04.005

[24] Gonçalves CA, Silva NL, Mauro MO, David N, Cunha-Laura AL, Auharek AS, et al Evaluation of mutagenic, teratogenic, and immunomodulatory effects of *Annona nutans* hydromethanolic fraction on pregnant mice. Genet Mol Res. 2014; 13:4392–4405. doi: 10.4238/2014.June.11.3 25036345 10.4238/2014.June.11.3

[25] Oliveira RJ, Mantovani MS, Pesarini JR, Mauro MO, da Silva AF, Souza TR, et al 6-Dimethylaminopurine and cyclohexamide are mutagenic and alter reproductive performance and intrauterine development *in vivo*. Genet Mol Res. 2015; 14:834–849. doi: 10.4238/2015.February.2.8 25730023 10.4238/2015.February.2.8

[26] Damasceno DC, Kempinas WG, Volpato GT, Consonni M, Rudge MVC, Paumgartten FJR. Anomalias congênitas: estudos experimentais. 2008; Editora Média, Belo Horizonte.

[27] Cunha-Laura AL, Oliveira RJ, Barros ALC DE, Siqueira JM DE, Vieira M DO C, Auharek SA. Maternal exposure to Cochlospermum regium: a toxicological evaluation. Rev Bras Farmacogn. 2013; 23:374–378. doi: 10.1590/S0102-695X2013005000005

[28] Auharek SA, Do Carmo Vieira M, Cardoso CAL, Oliveira RJ, Cunha-Laura AL. Reproductive toxicity of Campomanesia xanthocarpa (Berg.) in female Wistar rats. J Ethnopharmacol. 2013; 148:341–343. doi: 10.1016/j.jep.2013.04.010 23603192 10.1016/j.jep.2013.04.010

[29] OECD—Guidelines for Testing of Chemicals Section 4: Health Effects. 2001; Test No. 414: Prenatal Development Toxicity Study.

[30] OECD- Guidelines for Testing of Chemicals. Draft Proposal for an Extended One-Generation Reproductive Toxicity Study. 2009; No.28.

[31] Hoerauf KH, Schrögendorfer KF, Wiesner G, Gruber M, Spacek A, Kress HG, et al Sister chromatid exchange in human lymphocytes exposed to isoflurane and nitrous oxide in vitro. Br J Anaesth. 1999; 82:268–270. 10365006 10.1093/bja/82.2.268

[32] Heine J, Jaeger K, Osthaus A, Weingaertner N, Münte S, Piepenbrock S, et al Anaesthesia with propofol decreases FMLP‐induced neutrophil respiratory burst but not phagocytosis compared with isoflurane. Br J Anaesth. 2000; 85:424–430. 11103185 10.1093/bja/85.3.424

[33] Kotani N, Hashimoto H, Sessler DI, Atsuhiro K, Suzuki A, Takahashi S, et al Intraoperative modulation of alveolar macrophage function during isoflurane and propofol anesthesia. Anesthesiology. 1998; 89:1125–1132. 9822000 10.1097/00000542-199811000-00012

[34] Souza KM de. Avaliação de danos no material genético em anestesiologistas. 2016.

[35] Barrow MV, Taylor WJ. A rapid method for detecting malformation in rat fetuse. J Morphol. 1969; 127:291–305. doi: 10.1002/jmor.1051270303 4388962 10.1002/jmor.1051270303

[36] Wilson JG. Methods for administering agentes and detecting malformations in experimental animals In: WILSON J.G.; WAEKANY J. (Eds.). Teratology: Principles snd Thecniques. 1965; The University of Chicago Press, Chicago.

[37] Taylor P. Pratical Teratology. 1986; Academic Press, New York.

[38] Manson JM, Kang YJ. Test methods for assessing female reproductive and developmental toxicology 1994; In: Hayes A.W. (Ed). Principles and methods of Toxicology. Raven Press, New York.

[39] Staples RE, Schenell VL. Refinements in rapid clearing technic in the KOH-alizarin red method for fetal bone. Stain Technol. 1964; 39-61–63. 14106473

[40] Hayashi M, Morita T, Kodama Y, Sofundi T, Ishidate Junior M. The micronucleus assay with mouse peripheral blood reticulocytes using acridine Orange-coated slides. Mutat.Res. 1990; 245: 245–249. 1702516 10.1016/0165-7992(90)90153-b

[41] Julis J, Bartlett SA, Baarder S. Selective ethenolysis and oestrogenicity of compounds from cashew nut shell liquid. Green Chem. 2014; 16:2846–56. doi: 10.1039/C4GC00111G

[42] Rodrigues FHA, França FCF, Souza JRR, Souza JRR, Ricardo NMPS, Feitosa JPA. Comparison between physico-chemical properties of the technical cashew nut shell liquid (CNSL) and those natural extracted from solvent and pressing. Polímeros. 2011; 21:156–60. doi: 10.1590/S0104-14282011005000028

[43] Oliveira MSC, Morais MS, Magalhães DV, Batista WP, Vieira IG, Craveiro AA, et al Antioxidant, larvicidal and antiacetylcholinesterase activities of cashew nut shell liquid constituents. Acta Trop. 2011; 117:165–170. doi: 10.1016/j.actatropica.2010.08.003 20707981 10.1016/j.actatropica.2010.08.003

[44] Jeronimo CE, Nascimento LP, Balbino CP. Impacto Ambiental Derivado das Ações de Controle e Combate a Dengue no Rio Grande do Norte. Monografias Ambientais REMOA/UFSM. 2012; 9:2021–2030. Avaiable from: http://cascavel.cpd.ufsm.br/revistas/ojs-2.2.2/index.php/remoa/article/view/5914/3903. Cited 11 june, 2015.

[45] WHO- Recommended Classification of Pesticides by Hazard. Guidelines to classification. 2000–2002. Avaiable from: http://www.who.int/ipcs/publications/en/pesticides_hazard.pdf. Cited 3 February, 2016.

[46] Brasil—Ministério da Saúde. Orientações técnicas para utilização do larvicida pyriproxyfen (0,5G) no controle de Aedes aegypti. 2014; Avaiable from: http://u.saude.gov.br/images/pdf/2014/maio/30/Instrucoes-para-uso-de-pyriproxifen-maio-2014.pdf. Cited 3 February, 2016.

[47] Fares RC, Souza KP, Añez G, Rios M. Epidemiological Scenario of Dengue in Brazil. Biomed Res Int. 2015; 2015:321873 doi: 10.1155/2015/321873 26413514 10.1155/2015/321873 PMC4568054

[48] Gardner J, Rudd PA, Prow NA, Belarbi E, Roques P, Larcher T, et al Chikungunya Virus in the Saliva of Mice, Monkeys and Humans. PLoS One. 2015; 10:e0139481 doi: 10.1371/journal.pone.0139481 26447467 10.1371/journal.pone.0139481 PMC4598147

[49] Mathew G, Rahiman MA, Vijayalaxmi KK. *In vivo* genotoxic effects in mice of Metacid 50, an organophosphorus insecticide. Mutagenesis. 1990; 5:147–9. 2188066 10.1093/mutage/5.2.147

[50] Ni Z, Li S, Liu Y, Tang Y, Pang D. Induction of micronucleus by organophosphorus pesticides both *in vivo* and *in vitro*. Hua Xi Yi Ke Da Xue Xue Bao. 1993; 24:82–6. 8340099

[51] Aiub CA, Coelho EC, Sodré E, Pinto LF, Felzenszwalb I. Genotoxic evaluation of the organophosphorous pesticide temephos. Genet Mol Res. 2002; 1:159–66. 14963843

[52] Melo KM, Grisolia CK, Pieczarka JC, de Souza LR, Filho J de S, Nagamachi CY. FISH in micronucleus test demonstrates aneugenic action of rotenone in a common freshwater fish species, Nile tilapia (Oreochromis niloticus). Mutagenesis. 2014; 29:215–9. doi: 10.1093/mutage/geu005 24618992 10.1093/mutage/geu005

[53] Pereira BB, de Campos Júnior EO. Enzymatic Alterations and Genotoxic Effects Produced by Sublethal Concentrations of Organophosphorous Temephos in *Poecilia reticulata*. J Toxicol Environ Health A. 2015; 78:1033–7. doi: 10.1080/15287394.2015.1050566 26252754 10.1080/15287394.2015.1050566

[54] Carbajal-López Y, Gómez-Arroyo S, Villalobos-Pietrini R, Calderón-Segura ME, Martínez-Arroyo A. Biomonitoring of agricultural workers exposed to pesticide mixtures in Guerrero state, Mexico, with comet assay and micronucleus test. Environ Sci Pollut. Res Int. 2016; 23:2513–20. doi: 10.1007/s11356-015-5474-7 26423288 10.1007/s11356-015-5474-7

[55] Ghisi NdeC, Oliveira EC, Prioli AJ. Does exposure to glyphosate lead to an increase in the micronuclei frequency? A systematic and meta-analytic review. Chemosphere. 2016; 145:42–5. doi: 10.1016/j.chemosphere.2015.11.044 26688238 10.1016/j.chemosphere.2015.11.044

[56] Bretveld R, Brouwers M, Ebisch I, Roeleveld N. Influence of pesticides on male fertility. Scand J Work Environ Health. 2007; 33:13–28. 17353961 10.5271/sjweh.1060

[57] Barros AL, Cavalheiro GF, Souza AVM, Traesel GK, Franci JAA, Kassuya CAL, et al Subacute Toxicity Assessment of Difluobenzuron, an Insect Growth Regulator, in Adult Male Rats. Environ Toxicol. 2014; 31:407–14. doi: 10.1002/tox.22054 25266294 10.1002/tox.22054

[58] Thavara U, Tawatsin A, Chompoosri J, Bhakdeenuan P, Khamsawads C, Sangkitporn S, et al Comparative field efficacy of newly developed formulations of larvicides against *Aedes aegypti* (L.) (Diptera: Culicidae). Southeast Asian J Trop Med Public Health. 2013; 44:753–60. 24437310

[59] George L, Lenhart A, Toledo J, Lazaro A, Han WW, Velayudhan R, et al Community-Effectiveness of Temephos for Dengue Vector Control: A Systematic Literature Review. PLoS Negl Trop Dis. 2015; 9:e0004006 doi: 10.1371/journal.pntd.0004006 26371470 10.1371/journal.pntd.0004006 PMC4570708

[60] Evans L. Dias de falta ao trabalho por causa da dengue somam 1,8 mil. 2010. Avaiable from: http://www.em.com.br/app/noticia/gerais/2010/11/29/interna_gerais,195199/dias-de-falta-ao-trabalho-por-causa-da-dengue-somam-1-8-mi.shtml. Cited 1 February, 2016.

[61] Bilenky, T. Em três mese, INSS registra mais de 300 trabalhadores afastados por dengue. 2015. Avaiable from: http://www1.folha.uol.com.br/cotidiano/2015/04/1621308-em-3-meses-inss-registra-mais-de-300-trabalhadores-afastados-por-dengue.shtml. Cited 1 February, 2016.

[62] Brent RL. Reproductive and teratologic effects of low-frequency electromagnetic fields: a review of *in vivo* and *in vitro* studies using animal models. Teratology. 1999; 59:261–86. doi: 10.1002/(SICI)1096-9926(199904)59:4<261::AID-TERA12>3.0.CO;2-K 10331529 10.1002/(SICI)1096-9926(199904)59:4<261::AID-TERA12>3.0.CO;2-K

[63] Colet JM. Metabonomics in the preclinical and environmental toxicity field. Drug Discov Today, Technol. 2015; 13:3–10. doi: 10.1016/j.ddtec.2015.01.002 26190677 10.1016/j.ddtec.2015.01.002

[64] Neal A, Rountree AM, Philips CW, Kavanagh TJ, Williams DP, Newham P, et al Quantification of Low-Level Drug Effects Using Real-Time, *in vitro* Measurement of Oxygen Consumption Rate. Toxicol Sci. 2015; 148:594–602. doi: 10.1093/toxsci/kfv208 26396153 10.1093/toxsci/kfv208 PMC4830255

[65] OECD- Guidelines for Testing of Chemicals. Mammalian Erytrocyte Micronucleus Test. 1997; Test No. 474.

[66] OECD- Guidelines for Testing of Chemicals. In vitro mammalian Cell Micronecleus Test. 2012; Test. No. 487.

[67] Brasil—ANVISA—Agência Nacional de Vigilância Sanitária. Guia para a condução de estudos não clínicos de segurança necessários ao desenvolvimento de medicamentos. 2010; Brasília.

[68] Raheja KL, Jordan A, Fourcroy JL. Food and drug administration guidelines for reproductive toxicity testing. Reprod Toxicol. 1998; 2: 291–293. 10.1016/0890-6238(88)90034-2 2980359

[69] Zhang C, Xu D, Luo H, Lu J, Ping J, Wang H. Prenatal xenobiotic exposure and intrauterine hypothalamus-pituitary-adrenal axis programming alteration. Toxicology. 2014; 325:74–84. doi: 10.1016/j.tox.2014.08.015 25194749 10.1016/j.tox.2014.08.015

[70] Sally EOF, Anjos LA, Wahrlich V. Metabolismo Basal durante a gestação: revisão sistemática. Ciên. Saúde coletiva. 2013; 18 doi: 10.1590/S1413-81232013000200013 10.1590/s1413-81232013000200013 23358767

[71] Danesi R, Del Tacca M. Teratogenesis and immunosuppressive treatment. Transplant Proc. 2004; 36:705–7. doi: 10.1016/j.transproceed.2004.03.017 15110638 10.1016/j.transproceed.2004.03.017

[72] Yu Y, Yang Y, Zhao X, Liu X, Xue J, Zhang J, et al Exposure to the mixture oforganophosphorus pesticides is embryotoxic and teratogenic on gestational ratsduring the sensitive period. Environ Toxicol. 2017; 32 139–146. doi: 10.1002/tox.22219 26589364 10.1002/tox.22219

[73] Vargas MHM, Donadio MVF. Efeitos do estresse no período gestacional em diferentes modelos experimentais: uma revisão da literatura. Revista de Atenção a Saúde. 2014; 12: 81/41–86. doi: 10.13037/rbcs.vol12n41.2333

[74] Patil RD, Dwivedi P, Sharma AK. Critical period and minimum single oral dose of ochratoxin A for inducing developmental toxicity in pregnant Wistar rats. Reprod Toxicol. 2006; 22:679–87. doi: 10.1016/j.reprotox.2006.04.022 16781114 10.1016/j.reprotox.2006.04.022

[75] Oliveira RJ, Pesarini JR, Mauro MO, Fronza LS, Victorelli SG, Cantero WB, et al Effects of phenylalanine on reproductive performance and teratogenesis in mice. Genet Mol Res. 2014; 13:5606–16. doi: 10.4238/2014.July.25.16 25117318 10.4238/2014.July.25.16

[76] Eriksson UJ, Dahbstrom E, Larsson KS, Hellertron C. Increased incidence of congenital malformations in the offspring of diabetics rats and their prevention by maternalinsulin teraphy. Diabetes. 1982; 3: 1–6. 10.2337/diab.31.1.1 6759206

[77] Eriksson RSM, Thumberg L, Eriksson UJ. Effectts of interrupted insulin treatment on fetal outcome of pregnant diabetic rats. Diabetes; 1989; 38: 764–72. 2656344 10.2337/diab.38.6.764

